# Clinical and Genetic Spectrum of a Large Cohort With Total and Sub-total Complement Deficiencies

**DOI:** 10.3389/fimmu.2019.01936

**Published:** 2019-08-08

**Authors:** Carine El Sissy, Jérémie Rosain, Paula Vieira-Martins, Pauline Bordereau, Aurélia Gruber, Magali Devriese, Loïc de Pontual, Muhamed-Kheir Taha, Claire Fieschi, Capucine Picard, Véronique Frémeaux-Bacchi

**Affiliations:** ^1^Assistance Publique – Hôpitaux de Paris (AP-HP), Laboratoire d'Immunologie, Hôpital Européen Georges-Pompidou, Paris, France; ^2^Paris University, INSERM UMR1163, Imagine Institute, Paris, France; ^3^Study Center for Primary Immunodeficiencies (AP-HP), Hôpital Necker-Enfants maladies Hospital, Paris, France; ^4^Pediatrics Department, Jean Verdier Hospital, Assistance Publique des Hôpitaux de Paris, Paris 13 University, Bondy, France; ^5^Invasive Bacterial Infection and National Reference Center for Meningococci, Pasteur Institut, Paris, France; ^6^Department of Clinical Immunology, Hôpital Saint-Louis, Assistance Publique – Hôpitaux de Paris (AP-HP), Paris, France; ^7^Inserm U1126, Centre Hayem, Hôpital Saint-Louis, Paris, France; ^8^Centre de Recherche des Cordeliers, INSERM, Sorbonne Université, USPC, Université Paris Descartes, Complement and Diseases Team, Paris, France

**Keywords:** complement, genetic variants, meningococcal infections, deficiency, auto immune diseases

## Abstract

The complement system is crucial for defense against pathogens and the removal of dying cells or immune complexes. Thus, clinical indications for possible complete complement deficiencies include, among others, recurrent mild or serious bacterial infections as well as autoimmune diseases (AID). The diagnostic approach includes functional activity measurements of the classical (CH50) and alternative pathway (AP50) and the determination of the C3 and C4 levels, followed by the quantitative analysis of individual components or regulators. When biochemical analysis reveals the causal abnormality of the complement deficiency (CD), molecular mechanisms remains frequently undetermined. Here, using direct sequencing analysis of the coding region we report the pathogenic variants spectrum that underlie the total or subtotal complement deficiency in 212 patients. We identified 107 different hemizygous, homozygous, or compound heterozygous pathogenic variants in 14 complement genes [*C1Q*β (*n* = 1), *C1r* (*n* = 3), *C1s* (*n* = 2), *C2* (*n* = 12), *C3* (*n* = 5), C5 (*n* = 12), *C6* (*n* = 9), *C7* (*n* = 17), *C8* β (*n* = 7), *C9* (*n* = 3), *CFH* (*n* = 7), *CFI* (*n* = 18), *CFP* (*n* = 10), *CFD* (*n* = 2)]. Molecular analysis identified 17 recurrent pathogenic variants in 6 genes (*C2, CFH, C5, C6, C7*, and *C8*). More than half of the pathogenic variants identified in unrelated patients were also found in healthy controls from the same geographic area. Our study confirms the strong association of meningococcal infections with terminal pathway deficiency and highlights the risk of pneumococcal and auto-immune diseases in the classical and alternative pathways. Results from this large genetic investigation provide evidence of a restricted number of molecular mechanisms leading to complement deficiency and describe the clinical potential adverse events of anti-complement therapy.

## Key Points

– Restricted number of molecular mechanisms leading to complement deficiency.
– meningococcal or pneumococcal infections and auto-immune diseases are adverse events of anti-complement therapy.

## Introduction

The complement system is a key component of innate immunity and contributes to the elimination of pathogens, dying host cells and abnormal molecular structures (1, 2). Therefore, complement deficiencies disorders impair the immune system's ability to defend the body against foreign or abnormal cells that invade or attack it (3–5). Severe or multiple infections—mainly meningococcal infections—or severe autoimmune diseases (AID) in particular with childhood onset are the main features associated with complement deficiency (CD) (6–10). The complement is composed of a complex proteolytic cascade of more than 40 soluble and membranous proteins that interact in a various range of function. Inactive circulating complement proteins can be rapidly activated in a cascade fashion by three different activation pathways leading to a common terminal pathway. The classical pathway (CP) that involves the C1qrs complex, C2, and C4 is initiated by the binding of C1q to immune complexes. The activation of the lectin pathway (LP) is mediated by five pattern recognition molecules [as mannose binding lectin (MBL), ficolins, and collectins] that bind carbohydrate on pathogen surface and the MBL-associated serine proteases (MASP) (11). In contrast, the alternative pathway (AP) patrols for pathogen invasion with the constantly generation of the fluid phase C3 convertase complex C3(H2O)Bb formed with the spontaneous hydrolysis of C3, Factor B (FB), and Factor D (FD) that cleaves C3 at low level. Healthy host cells are protected against complement attacks by many regulation factors such as Factor H (FH) and Factor I (FI) in the plasma, CD46, and CD55 on cells and are thus resistant to damage of the low-grade activation (12). On pathogens that lack specific regulators of complement, C3b interacts with FB to form a surface-bound AP C3 convertase (C3bBb). Properdin (FP), a key positive regulator of the AP is required for an efficient C3b feedback loop and amplified AP activation (3). These three different pathways lead to the formation of C3 convertases (alternative and classical C3 convertases) which cleave C3 in the bioactive opsonin C3b and C3a following the rapid formation of the C5 convertase which cleaves C5 into C5a and C5b and induces the first step of the membrane attack complex (MAC) formation. The MAC assembly requires the sequential and irreversible association of complement components C5b, C6, C7, C8 (C8β and C8αγ), and C9. Therefore, the activation of the complement system induces opsonization of C3b and degraded fragments (iC3b/C3dg) of C3 on bacterial surface promoting phagocytosis and the capacity to kill pathogens by the MAC (13). The anaphylatoxins, C3a, and C5a are constantly released during complement activation and induce an inflammatory response (1).

CD are inherited as autosomal recessive traits, except for properdin deficiency that is X-linked recessive and C1 inhibitor deficiency that is inherited as an autosomal dominant pattern. If the MBL deficiency is common, CD are generally rare, with an estimated prevalence of 5% of all primary immunodeficiencies (7). Published data of CD are limited to single case reports (14–19), small case series (20, 21), and few multicenter studies (9, 22, 23), evaluating clinical manifestations. Here, we report the immunological, genetic, and clinical features of the largest worldwide cohort ever described of 212 patients with total or sub-total complement deficiencies.

## Methods

### Patients

Patients addressed for complement testing often present, amongst others, recurrent microbial infections, unexplained inflammation, auto immune disease (AID), or renal diseases. Patients included in this study came from different French departments of medicine. Complement deficient patients (273 index cases and 48 asymptomatic related individuals) diagnosed between 1988 and 2018 were reported in this study including 75 previously published patients (9, 15, 20, 24–26). Clinical data were available for 84% of the cohort (*n* = 230) (Table 1). Genetic screening was possible for 212 patients.

**Table 1:** Clinical characteristics of patients with complement deficiency.

	**Total**	**Classical pathway deficiencies (*****n*** **=** **73)**			**Alternative pathway and C3 deficiencies(*****n*** **=** **46)**							**Terminal pathway deficiencies (*****n*** **=** **154)**					
				**Total**	**Decreased C3 (*****n*** **=** **34)**			**Total**	**Normal C3 (*****n*** **=** **12)**		**Total**						**Total**
**Proteins**		**C1**	**C2**		**FH**	**FI**	**C3**		**FP**	**FD**		**C5**	**C6**	**C7**	**C8**	**C9**	
Patients, *n* (%)	273	6 (1 C1q, 3 C1r, 2 C1s)	67	73/273 (27)	13	17	4	34/273 (12)	11	1	12/273 (4)	21	57	45	26	5	154/273 (56)
Male *n* (%)	148 (54)	3 (50)	29 (43)	32/73 (44)	7 (54)	8 (47)	3 (75)	18/34 (53)	11 (100)	1	12/12 (100)	12 (57)	35 (61)	24 (53)	14 (54)	1 (20)	86/154 (56)
Median age at diagnosis (Quartile1–Quartile3)	15 (7–20)	7 (5–8)	20 (4–40)		0 (0–1)	7 (3–13)	9 (7–11)		14 (9–17)	7		16 (11–23)	18 (13–29)	21 (16–27)	20 (11–43)	25 (12–43)	
Available Clinical data, *n* (%)	230	6 (100)	45 (67)	51/73 (70)	13 (100)	15 (88)	4 (100)	32/34 (94)	11	1	12/12 (100)	18 (85,7)	50 (87,7)	40 (88,9)	22 (84,6)	5 (100)	135/154 (88)
Infectious disease, *n* (%)	155	1 (16, 7)	26 (58)	27/51 (53)	0	13/15 (87)	4 (100)	17/32 (53)	11 (100)	1 (100)	12/12 (100)	16/18 (89)	33/50 (66)	33/40 (73)	17/22 (77)	1/5 (20)	100/135 (74)
Meningococcal disease, *n* (%)	113	0	2/26 (8)	2/27 (7)	0	2/13 (15)	0	2/17 (12)	11 (100)	1 (100)	12/12 (100)	15 (93,8)	33/33 (100)	31 (93,9)	17 (100)	1 100)	97/100
Pneumococcal infection, *n* (%)	24	1 (100)	14/26 (54)	15/27 (55)	0	4/13 (31)	4 (100)	8/17 (47)	0	0	0	0	0	0	0	0	0
Other infection, *n* (%)	20	0	10/26 (38)	10/27 (37)	0	7/13 (54)	0	7/17 (41)	0	0	0	1 (6, 2)	0	2 (6, 1)	0	0	3/100
Auto Immune disease, *n* (%)	33	5 (83,3)	11 (24)	16/51 (31)	0	1/15 (7)	0	1/32 (3)	0	0	0	1/18 (6)	10/50 (20)	1/40 (3)	1/22 (5)	3/5 (60)	16/135 (12)
Other disease (including kidney diseases), *n* (%)	38	0	8 (18)	8/51 (16)	13 (100)	1 (7)	0	14/32 (44)	0	0	0	1/18 (6)	7/50 (14)	6/40 (15)	2/22 (9)	0	16/135 (12)
Asymptomatic, *n* (%)	3	0	0	0	0	0	0	0	0	0	0	0	0	0	2/22 (9)	1 (20)	3/135 (2)
Familial screening (not included in patients)	64	0	7	7	0	1	0	1	8	0	8	5	15	7	5	0	32
Available genetic data, *n* (%)	212	6 (100)	64(100)	70/73 (95)	13 (100)	15 (88)	4 (100)	32/34 (94)	10 (90,9)	1 (100)	11/12 (92)	17/21 (68)	39/57 (68)	29/45 (64)	12/26 (46)	2/5 (40)	99/154 (64)

### Complement Assays

A functional complement activity assay of the classical (CH50) and alternative pathway (AP50) hemolytic activities were determined according to standard procedures (27). CH50 and AP50 are based on lysis of Ab-sensitized sheep and rabbit erythrocytes, respectively. To get complexion of the divalent cations calcium and magnesium needed for the activation of the classical pathway, and thus to avoid *in vitro* complement activation, blood was drawn directly into EDTA-containing tubes. The addition of Veronal-buffered saline (1.8 mM sodium barbiturate, 3.1 mM barbituric acid, and 0.15 M NaCl, pH 7.4, containing 0.75 mM Ca^2+^ and 2.5 mM Mg^2+^) restores the hemolytic function of EDTA-plasma samples. According to CH50 and AP50 results, further assays are performed to determine the missing complement component. The quantification of individual complement factors by ELISA or hemolytic assays may uncover quantitative or functional defects in protein. In routine diagnosis, C3 and C4 plasma levels are measured by nephelometry (Siemens, Inc., Newark, DE). Levels of classical complement components (C1q, C1r, C1s), of alternative pathway (FH, FI, FP, FD, FB) and of terminal pathway components (C5, C6, C7, C8, C9) were measured by home-made enzyme-linked immunosorbent assay (ELISA) [antibodies are described in Supplemental Table 1 and in (15, 24, 28)]. Hemolytic FH and FI assays were used to diagnose functional FH and FI deficiencies, respectively. CH50, AP50, and ELISA assays were performed on plasma from EDTA whole blood (plasma EDTA). Results were expressed as a percentage of the mean result obtained on a reference plasma EDTA pool obtained from 190 healthy donors.

### Genomic DNA Sequencing

Genetic testing was performed on 212/273 patients (77.7%). Genomic DNA was extracted and purified from whole blood using standard procedures. Screening of type 1 C2 deficiency was performed by polymerase chain reaction (PCR) and gel migration on agarose as previously described (29). Amplifications by PCR (GoTaq DNA Polymerase, Promega, USA) of all exons and flanking splices sites of all the complement components were performed using specific forward and reverse primers (available on request). PCR products were purified (Multiscreen plates, Millipore, USA) and sequenced by the Big Dye terminator cycle sequencing methods using the same forward and reverse primers that for amplification (QiaQuick PCR Purification Kit, Qiagen SA, Hilden, Germany). Sequence analyses were then conducted (Applied Biosystems, Waltham, Massachusetts, USA) and read using Sequencher 5.0 (Gene Codes Corporation, USA). The interpretation of mutations was performed by comparing sequences of patients with the reference sequences exons using Alamut 2.3 (Interactive Biosoftware, France).

### Ethics

This study was carried out in accordance with the recommendations of French Ethics Committee (“Comité de Protection des Personnes Ile de France 5,” reference a-11–15, number 1922081) with written informed consent from all subjects.

## Results

### Screening Strategy

CH50 and AP50 were used to screen deficiency in the complement pathways (30). Classical pathway deficiency (CPD) was suggested by a decreased CH50 activity (below 10% of normal values) with normal AP50 activity. Terminal pathway deficiency (TPD) was defined by undetectable CH50 and AP50 activities (below 10% of normal values). The addition of normal plasma (8 μL) to the complement deficient plasma restored its ability to sustain CH50 activity, whereas the addition of plasma depleted of the deficient component did not. Patients with total TPD had undetectable CH50 and AP50, patients with sub-total deficiencies (SD) in C5, C6, or C7 had a low but detectable CH50 activity. Normal CH50 activity and an undetectable AP50 activity with a normal C3 level suggest a deficiency in one of the alternative pathway components (i.e., FB, FD, or FP). Significantly isolated reduction of C3 can reveal C3, FI or FH deficiencies (C3D, CFID, and CFHD). Quantitative or functional measurements of individual complement components followed by the screening of the all coding regions of the identified genes lead to the final diagnosis.

We diagnosed 273 patients with CD including 73 (27%) patients with CPD, 46 (17%) with AP deficiency (APD), and 154 (56%) with TPD. Patients with APD were divided into patients with low plasma C3 level (C3, Factor I and Factor H deficiencies, *n* = 34) or normal C3 level (Properdin and Factor D deficiencies, *n* = 12).

Applying sequencing analysis we identified 107 pathogenic variants in 14 complement genes (Supplemental Table 2 and Supplemental Figures 1–3).

### Patients' Characteristics

The median age at diagnosis was 15 (Q1–Q3:7–20). A total of 148 patients were male (148/273, 54.2%). Clinical data were available for 230 patients (230/273, 84.2%). Patients suffered from infectious diseases (*n* = 155/230, 67.3%), auto-immune disease (*n* = 33, 14.3%), kidney disease [atypical Hemolytic Uremic Syndrome (aHUS) or C3 Glomerulopathy] (*n* = 14, 6%). The type of infections was mostly meningococcal (*n* = 113, 72.9%) or pneumococcal (*n* = 24, 15.5%) (Table 1).

#### Genetic and Clinical Characteristic of CPD

We identified 73 patients (73/273; 27%) with an undetectable CH50 activity associated with a low level of one of the components of the C1qrs complex (C1q: *n* = 1, C1r: *n* = 3, C1s: *n* = 2) or C2 protein (*n* = 67). Clinical data and molecular characterization of the genetic defects were available in 51 (51/73, 69.8%) and 73 (100%) patients, respectively.

Median age of diagnosis was 26 years; the sex ratio (M/F) was 0.44. A total of 27 and 16 patients were diagnosed after infections (*n* = 27/51; 52.9%) including *Streptococcus pneumoniae* in 53.8% or with Systemic Lupus Erythematosus (SLE) (16/51; 31.4%), respectively.

C2 deficiency (C2D) was confirmed in 67 patients from 67 kindred. Median age of diagnosis was 20 years; the sex ratio (M/F) was 0.43. Half of C2D patients (26/45, 57.8%) presented with recurrent or severe infections mostly due to *S. pneumoniae* (*n* = 14, 53.8%). Two patients (7.7%) presented with meningococcal meningitis. Other clinical manifestations in C2 deficient patients included AID (*n* = 11, 24.4%), abnormalities in serum immunoglobulin concentrations and various clinical manifestations such as renal, pulmonary or cutaneous manifestations (Table 1).

Genetic analysis revealed that 54 patients carried the homozygous 28 base-pairs deletion (Del28pb) in the *C2* gene. Compound heterozygosity (heterozygous Del28pb combined with another rare variant) or two heterozygous rare variants was identified in 9 and 1 patients, respectively. The 12 molecular defects are small deletion or insertion (*n* = 3), non-sense variant (*n* = 2), canonical splice sites (*n* = 2) and missense variant (*n* = 5) (Table 2, Supplemental Figure 1).

**Table 2:** Variants spectrum associated with quantitative and functional deficiency.

**Genes**	**C1**	**C2**	**CFH**	**CFI**	**C3**	**CFP**	**CFD**	**C5**	**C6**	**C7**	**C8**	**C9**
Variant, *n* (Hmz patients/Htz patients)	6 (5/1)	12 (54/10)	7 (13/0)	18 (9/6)	5 (3/1)	10	2 (0/1)	12 (11/6)	9 (19/20)	17 (15/14)	7 (7/5)	3 (1/1)
Missense variant	2	5	4	10	0	5	1	5	1	5	1	0
Nonsense variant	3	2	1	4	2	2	0	3	3	2	4	2
Insertions/duplication/deletion	0	3	1	2	2	3	1	2	3	6	1	0
Splice sites changes	1	2	1	2	1	0	0	2	2	4	1	1
Previously identified in GnomAD	1	5	2	9	1	3	0	8	8	11	7	3
Novel	5	7	5	9	4	7	2	4	1	6	0	0

Six unrelated patients were diagnosed with a complete deficiency of one of the C1qrs complex component. Five patients presented pediatric onset of AID and one had recurrent ear, nose and throat infections. The median age at diagnosis was 7 years (Q1–Q3: 5–8), with a sex ratio (M/F) of 2 (Table 1). Six very rare genetic changes with minor allele frequency <0.0001% were identified in the coding exons of the C1q (*n* = 1), C1r (*n* = 3), and C1s (*n* = 2). Five patients had homozygous variants (1 in *C1QB*, 2 in *C1R*, and 2 in *C1s*) and one C1r deficient patient carried two *C1R* heterozygous variants (Table 2, Supplemental Figure 1).

#### Genetic and Clinical Characteristic of APD With Low C3 Level

Among the AP deficient subjects, 34 (12.5% of the whole cohort) suffered from FI (*n* = 17), FH (*n* = 13), or C3 (*n* = 4) deficiency.

Among the 17 patients with FI deficiency, 9 were females (52.9%) and the median age at diagnosis was 7 years. Clinical and molecular mechanisms were available in 15 patients. Thirteen patients (13/15; 86.7%) presented with meningococcal disease (*n* = 2), *S. pneumoniae* infection (*n* = 4) and recurrent respiratory infections (*n* = 7). The FI level was within the normal range (*n* = 1), below the normal range (*n* = 2) and undetectable (*n* = 13). In all patients' plasma FH was below the normal ranges.

**Figure 1:**
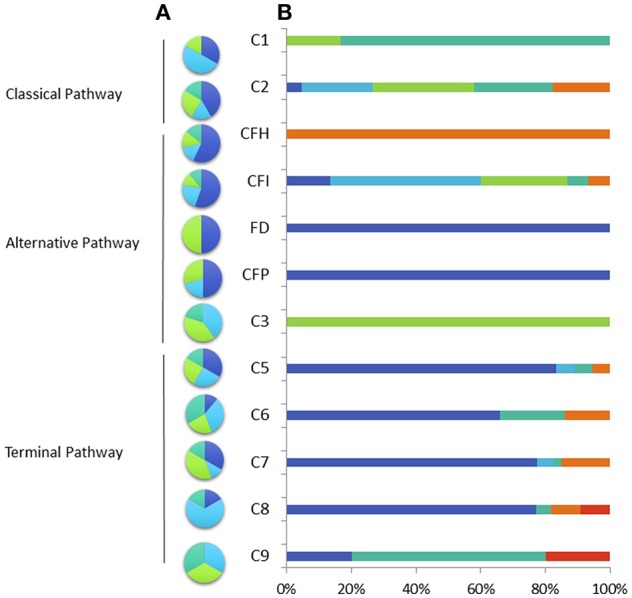
Percentage of variant type **(A)** nonsense (light blue), missense (dark blue), deletion or insertion (green), splice site mutation (blue green) and clinical phenotype **(B)** in the French cohort of patients with total and subtotal deficiency. Invasive meningococcal disease (dark blue), other infection (light blue), auto-immune disease (blue green), renal disease (orange), pneumococcal infection (green) other affection (red).

Patients carry two copies of the same rare variant (*n* = 9) or two rare variants (*n* = 6) (Table 2, Figure 1, Supplemental Figure 2). The molecular basis was missense (*n* = 10), nonsense (*n* = 4) variants, insertions/duplication (*n* = 2) or splice sites changes (*n* = 2) (Supplemental Figure 2). Nine out the 17 variants were referenced with a minor allele frequency below 0.01 and 8 were not reported. All were predicted to be pathogenic using Polyphen-2 (data not shown). Two variants (p.Ile340Thr; p.Asp524Val) had no effect on FI synthesis but caused a defect of FI activity (not effective in inactivating membrane-bound C3b) (31, 32). Thirteen unrelated patients were diagnosed with a complete FH deficiency. All patients had low C3 levels secondary to alternative pathway consumption (Min–Max: 40–234 mg/L). Mean CH50 activity of these patients was 33% (<10 to 79%). FH level was below 0.1% of normal values in 4 cases and comprised between 2 and 12% of normal values in 8 cases. One patient had a normal FH level (data not shown).

The age at diagnosis was below 1 year in 10 out the 13 cases. All patients had a renal disease, 10 patients presented with atypical aHUS (76.9%) and 3 with C3 glomerulopathy (C3G) (23.1%). All patients presented variants at the homozygous state (Table 2, Supplemental Figure 2). Half of the cases carry the small deletion (c.3693_3696delATAG p.^*^1232Ilefs^*^38) and were from North African descends.

Four unrelated patients were found to have a complete deficiency in C3 (Table 1). All presented with *S. pneumoniae* infections (Table 1, Figure 1). Three patients presented with homozygous variants and one was compound heterozygous (Supplemental Figure 2).

#### Genetic and Clinical Characteristic of APD With Normal C3 Level

Among the AP deficient subjects, 12 (4.4% of the whole cohort) presented FP deficiency (*n* = 11) or FD deficiency (*n* = 1). All these patients presented with *Neisseria meningitidis* infections.

X-linked FP deficiency was identified in 11 male patients from 11 unrelated families including 9 cases of FP type 1 deficiency (FP levels below 0.3 μg/ml) and 2 cases of FP type II deficiency (FP level between 2 and 5 μg/ml). Median age of diagnosis was 14 years, ranging from 1 to 23 years. Eight asymptomatic family members were diagnosed when the familial segregation was performed. The screening of the molecular genetic was performed in 10 out the 11 pedigrees. The genetic changes were missense variants (*n* = 5) non-sense variant (*n* = 2), or small deletion resulting in frameshift (*n* = 3) (Table 2, Supplemental Figure 2).

We here report the fifth patient with a complete FD deficiency (17, 33–35) (Supplemental Figure 4A). The patient presented at 3 years old a *purpura fulminans* successfully treated by cefotaxime. Diagnosis of *N. meningitidis* infection was confirmed by PCR on cerebrospinal fluid which identified a group B strain from clonal complex 41/44 (B:41/44). Assessment of the complement system was performed at the age of 7 years old. CH50 activity was normal but he had undetectable AP50 activity (<10%). FP and FB levels were normal. Addition of purified FD (CompTech) at the physiological concentration of 2 μg/ml restored the AP50 activity (Supplemental Figure 4B). Genetic study of *CFD* identified one heterozygous frameshift mutation (c.677–678delinsTTCT) and one heterozygous missense rare variant (c.653T>C, p.Leu218Pro) (Supplemental Figure 4C). Each allele was found in one parent, allowing to conclude that the alleles are on different chromosomes. The Leu218 residue is buried in a beta-strand (Supplemental Figure 4D) and is located between the p.Ser208 directly involved in the serine-protease activity of FD and the self-inhibitory loop of FD (p.Val221 to p.Val227, Supplemental Figure 4E).

#### Genetic and Clinical Characteristic of TPD

A total of 139 patients were diagnosed with complete TPD: C5D (*n* = 20, 14%), C6D (*n* = 52, 36.8%), C7D (*n* = 37, 26.2%), C8D (*n* = 26, 18.4 %), C9D (*n* = 5; 3.5%), and 15 with SD: C5SD (*n* = 1/15, 7%), C6SD (*n* = 4, 27%), C7SD (*n* = 10, 66%). Patients with available clinical data (*n* = 135) presented with *N. meningitidis* infections in 72% (*n* = 97/135) or with AI diseases in 12% (*n* = 16/135). The screening of the genetic abnormalities was performed on 99 patients (99/154 = 64%). A total of 53 and 47 patients carried homozygous variants (53/99 = 53%) or two heterozygous (47/99 = 47%) variants.

Pathogenic variants were identified in the coding exons or flanking splice sites of the C5 (*n* = 11), C6 (*n* = 9), C7 (*n* = 17), C8 b chain (*n* = 7), and C9 (*n* = 3) (Figure 1, Supplemental Figure 3). The pathogenic variants were missense (*n* = 11), nonsense (*n* = 14), small deletions (*n* = 12) or located in splice sites (*n* = 10). We identified 15 recurrent mutations accounted for 78% of the pathogenic alleles (153/197 alleles) (Table 3). The variant allele frequencies ranged from 0.0008 to 0.23% and are variable according to the geographical origin. Four of these variants: (c.960_962delCAA p.Asn320del in *C5* gene; c.1879delG p.Asp627Thrfs^*^4 in *C6* gene; c.1135G>C p.Gly379Arg in the *C7* gene, and c.1282C>T p.Arg428^*^ in *C8B* gene) accounted for 41% of the molecular defects (80/197).

**Table 3:** Recurrent Pathogenic rare variant identified in the cohort.

**Complement component**	**Recurrent pathogenic variants**	**GnomAD (allele frequency, %)**	**CD alleles, *n***	**%**	**VAF Europe (non-Finnish) (%)**	**VAF Africa (%)**
C5	Exon 9 c.960_962delCAA	0.002840	16	45,7	0.002333	0.004013
(*n =* 17)	Exon 07 c.713T>C p.Ile238Thr	0.007323	5	14,3	0.006104	0.000
	Exon 01 c.55C>T p.Gln19^*^	0.01486	3	8,6	0.000	0.1282
	Exon 07 c.754G>A p.Ala252Thr (C5SD)	0.05239	2	5,7	0.000	0.5281
	Other	–	8	23,5	–	–
C6	Exon 13 c.1879delG p.Asp627Thrfs^*^4	0.1089	38	48,7	0.0007778	0.1134
(*n =* 39)	Exon 08 c.1138delC p.Gln380Serfs^*^7	0.06907	14	17,9	0.002328	0.7292
	Exon 07 c.821delA p.Gln274Argfs^*^46	0,0492	11	14,1	0.02094	0.3926
	Exon 02 c.143G>A p.Arg48Lys	0.02159	5	6,4	0.03881	0.004005
	Intron 16 c.2381+2T>C (C6SD)	0.2218	5	6,4	0.3814	0.06809
	Other	–	5	6,4	–	–
C7	Other	–	22	37,9	–	–
(*n =* 29)	Exon 10 c.1135G>C p.Gly379Arg	0.01256	13	22,0	0.01877	0.000
	Exon 12 c.1561C>A p.Arg521Ser (C7SD)	0.2354	12	20,3	0.4135	0.07931
	Exon 17 IVS17+2 c.2350+2T>C	0.02832	11	18,6	0.04881	0.000
C8β	Exon 09 c.1282C>T p.Arg428^*^	0.1125	13	54,2	0.1892	0.03206
(*n =* 12)	Other	–	6	25,0	–	–
	Exon 06 c.850C>T p.Arg284^*^	0.002479	3	12,5	0.003106	0.000
	Exon 03 c.361C>T p.Arg121^*^	0.01353	2	8,3	0.01848	0.006152
C2	c.841_849+19del p. (Val281_Arg283del)	0.4764	117	91,4	0.7159	0.1002
(*n =* 64)	Other	–	11	8,4	–	–
FH	c.3693_3696 delATAG p.X1232I fsX38	novel	12	46,2	–	–
(*n =* 13)	Other	–	14	53,8	–	–

* *STOP codon*.

SD is defined by the presence of low but detectable and functional residual complement components. TP subtotal deficiency of C5 (C5 level: 2%), C6 (C6 level: 2%), and C7 (C7 level ranging from 0.1 to 1%) were found in 15 patients. The molecular alterations of the SD were the *C5* missense variant p.Ala252Thr, the *C6* splice site c.2381+2T>C variant and the *C7* missense variant p.Arg521Ser. Compound heterozygosity (combined SD and D alleles) or two SD variants were identified in 11 (C6SD/C6D, *n* = 3; C7SD/C7D, *n* = 8) and 4 patients (C5SD/C5SD, *n* = 1; C6SD/C6SD, *n* = 1; C7SD/C7SD, *n* = 2) respectively. The C5SD patient who carry the homozygous p.Ala252Thr variant presented with a normal AP50 hemolytic activity.

## Discussion

We here report the spectrum of complement genes pathogenic variants in the largest worldwide cohort of patients with subtotal or total complement deficiency.

Except for FP, we posited autosomal recessive transmission of the abnormal allele in these families and sought homozygous or compound heterozygous variants within the same gene. Variant frequency in the general population is a key criterion used in the clinical interpretation of sequence variants. As strong disease-predisposing alleles are likely to be deleterious, we focused on rare variants with a minor allele frequency below 0.1%. In our study a total of 48 variants (48/107, 45%) were not found in the Genome Aggregation Database (gnomAD) that has the ability to identified ultra-rare variant. However, 9 variants (9/48, 19%) not detected in gnomAD, have been already reported in patients with CD from unrelated families, including 5 variants (5/10, 50%) identified in FP deficient patients suggesting a common ancestral origin for these groups of families or a mutational hotspot (36–38).

Interestingly, we showed that half of the pathogenic variants identified in our cohort (59/107, 55%) were previously identified in healthy control but were overrepresented in CD patients. Deleterious variants in complement genes are rare, but the incidence varies considerably within populations. C7 deficiency caused by the pathogenic variant p.Gly379Arg is reported in 1% of the Israeli Moroccan Jewish population (39). C6 deficiency has a high prevalence in western Cape South Africans and in African Americans (40, 41) and the C5 variant p.Ala252Thr leading to subtotal C5 deficiency is prevalent in sub–Saharan Africa (22). In our study, we identified 15 recurrent deleterious variants in genes coding for the lytic complex. The pathogenic variant (c-960–962delCAA) found in half of the patients with C5 deficiency in our cohort has been identified in North-African populations, suggesting that this pathogenic allele may have a higher incidence within this restrictive geographic area (21, 42). Three changes of the *C7* gene (p.Gly379Arg, p.Arg521Ser, and 5′ splice donor site of intron 17) with a significantly higher frequency of the variant allele frequency in a European rather than in an African population account for 65.5% of the C7 molecular defects identified in our cohort (24, 39). Approximately 1% of Western European healthy individuals, exhibit the pathogenic allele in C2 gene caused by a 28-base pair genomic deletion. This deletion causes skipping of exon 6 during RNA splicing, resulting in generation of a premature termination codon (29). Our study showed that 90% of the patients with complete C2D carried this molecular defect. Altogether, we showed that half of the CD are explained by a restricted number of molecular abnormalities.

We identified 3 overexpressed variants in the *C6* gene (63/78 C6D alleles) reported with a higher frequency in healthy donor from the Black African communities with an estimated frequency ranging from 0.1 to 1% when compared to the European population (41). Many countries outside Africa have been a common destination for African immigrants over the years. All these “at risk variants” can be spread to other countries and might have arisen spontaneously in one allele in an individual many generations ago. The higher risk of invasive meningococcal disease (IMD) in patients with TPD have been extensively described (43). These patients usually develop IMD with bacterial isolates belonging to unusual phenotypes and genotypes (9). The high frequency of deficient alleles in individuals of African descent leading to IMD could impact medical care and genetic counseling in the general population. Indeed, a selective screening of the 3 most frequent pathogenic variants could be a reasonable option to identify C6–deficient individuals requiring long term protection. All these observations highly suggest that the identified variants are pathogenic and shows the growing trend to identifying pathogenic variants previously restricted to single, ethnically isolated regions in many different ethnic groups worldwide.

The majority of variants was non-sense (*n* = 28) or small deletion/insertion/duplication (*n* = 23) variants that possibly impaired transcription or translation. We identified 4 missense mutations leading to SD in C5 (p.Ala252Thr) (26), C7 (p.Arg521Ser) (20), and in FP (p.Glu244Lys and p.Arg102Trp (26). Interestingly, we identified 38 missense variants highly suggestive to be responsible for CD. Variants can either prevent synthesis of a protein resulting in quantitative deficiency or can lead to impaired protein synthesis resulting in functional deficiency. Three missense variants in *CFH* gene affect conserved cysteine residues characteristic of SCR modules and therefore block the normal secretion of the protein as previously demonstrated and explain the undetectable FH in patient's plasma (44). Three rare genetic variants in CFI (p.Val152Met, p.Gly162Asp, p.Ala258Thr) have been found in patients with age-macular degeneration and in aHUS and low serum FI antigenic levels (45). Our data confirm that these 3 variants impair the secretion and lead to FID. An original finding in this study is the identification of 4 functional deficient variants, one in *C1QB* (p.Gly90Ser) (25), one in *CFH* (p.Arg53Cys) (46), and two in *CFI* (p.Ile340Thr, p.Asp524Val) (47, 48). The structural and functional consequences of the rare missense variant remain unexplored in more than half of the newly identified variant. Bioinformatics prediction using structure function maps is useful to determine the likely consequences of the variants. All missense variants identified in the deficient patients are classified probably damaging by Polyphen-2 (Supplemental Table 3). Altogether, except in few cases, CD are secondary to quantitative complement deficiency assessed by the lack of detectable protein level. Further studies using recombinant proteins expression system are needed to confirm these hypotheses.

Recently, an increasing number of drugs targeting several complement proteins are being evaluated (49) and clinical data collected from studies could help predict the side effects of these complement-targeted drugs. Our study confirmed that only a restricted set of pyogenic bacteria, *N. meningitidis* and *S. pneumoniae* appear to be prominent in patients with CD. We identified 24 patients with documented pneumococcal infection, among whom 58.3% (*n* = 14) presented C2D, 16.7% (*n* = 4) complete C3D and 20.8% (*n* = 5) CFID, none presented with TPD. For the 2005–2015 period, the incidence rate of IMD in France ranged from 0.78 and 1.23 per 100.000 inhabitant with 5,772 cases of IMD that were reported, of which 4,090 (71%) of cases among <1 to 24 years old (mean of 409 cases per year for this population) (50). During our study period, the number of patients with TP deficiency has ranged from 2 to 5 cases per year. If considering the median age of complement deficiency diagnosis, around 1% of the patients that presented with IMD had a TP deficiency confirming the high risk of meningococcal infection in individuals without functional lytic complexes. Interestingly patients with TP, FD, and FP deficiencies are associated with IMD but C3D and FID patients suffered of pneumococcal infections. Although frequently asymptomatic, we confirmed that homozygous C2D are associated with severe infections and AID (23). Surprisingly half of the patients with C6D were found to have an AID but no IMD. As our series of patients is small and the analysis is based only on index cases, we cannot exclude a fortuitous association. Therefore, AID could develop as a side effect of treatment with new drugs targeting the C6 and should be monitored.

Current complement analysis goes far beyond the determination of CH50, C3, C4, and AP50 (30, 51). Abnormal test results were followed up by quantitative immunochemical measurements of individual complement components following by the screening of the all coding regions of the identified genes. MBL deficiency has been associated, although not conclusively, with increased susceptibility to several infections (52). However, alleles that confer MBL deficiency are common with a frequency up to 30%, which suggests a redundant role of the lectin pathway in host defense (53). Therefore, we do not perform the screening of MBL deficiency in the systematic exploration of the immunity in clinical practice.

Despite phenotypic differences, simple and routine exploration can reveal all CD, but the genetic characterization confirms the diagnosis. The presence of pathogenic variants frequent in the African population provides an opportunity to identified highly susceptible individuals for IMD. Our work supports the recommendation to systemically explore patients with IMD with atypical meningococcal isolates for CD and particularly for TPD. Our data provide strong support that complement inhibition confers high susceptibility to Neisseria and pneumococcal infections but also to AID and strategies to mitigate this risk are mandatory.

## Data Availability

The raw data supporting the conclusions of this manuscript will be made available by the authors, without undue reservation, to any qualified researcher.

## Author Contributions

Conception and design: VF-B. CE, JR, PV-M, PB, AG, and MD carried out the complement assessment and the sequencing analysis. Provision of study materials and patients: LdP, CF, CP, and M-KT. Data collection and assembly: VF-B and CE. Data analysis and interpretation: VF-B, CE, JR, M-KT, CF, and CP. Manuscript: VF-B and CE. Final approval of manuscript: all authors.

### Conflict of Interest Statement

VF-B received fees from Alexion Pharmaceuticals for invited lectures and/or board membership. VF-B is the recipient of a research grant from Alexion Pharmaceuticals. The remaining authors declare that the research was conducted in the absence of any commercial or financial relationships that could be construed as a potential conflict of interest.
