# Supplementary material for: Clinical and Genetic Spectrum of a Large Cohort With Total and Sub-total Complement Deficiencies
Source: Front Immunol. 2019 Aug 8;10:1936. doi: 10.3389/fimmu.2019.01936 (PMC6694794; doi:10.3389/fimmu.2019.01936)
Supplement: Supplementary file 1 [file Table_1.DOCX]

**Supplemental Materials**

**Supplemental Figure legends**

**Supplemental Figure 1. Pathogenic variants identified in patients with CP deficiency.**

Homozygous variants are numbered in black circles and heterozygous variants in white circles. Genes and introns are not at scale.

**Supplemental Figure 2. Pathogenic variants identified in patients with AP deficiency.** Homozygous variants are numbered in black circles and heterozygous variants in white circles. Genes and introns are not at scale.

**Supplemental Figure 3**. **Pathogenic variants identified in patients with TP deficiency.** Homozygous variants are numbered in black circles and heterozygous variants in white circles. Genes and introns are not at scale.

**Supplemental Figure 4.** Factor D deficiency

**A.**Pedigree of the family **B.** Restauration of AP50 activity in plasma from P1 by addition of purified CFD; HD = healthy donors **C.** Chromatograms of the *CFD* mutations in P1 (RefSeq : NM_001928) **D.** Localization of the p.L218P (red) missense mutation in the crystal structure of CFD (PDB: 2XWB[^E^](applewebdata://44702A4F-45BC-419B-8986-9F9DED401777/)^4^). **E.** Mutations associated with CFD deficiency. CFD is a 253 aa serin-protease with a self-inhibitory loop (dark green). The three residues involved in serine-protease activity are represented in blue.

**Supplemental Table 1 :** Antibodies used for home made ELISA

Complement antigen concentration was measured by a sensitive ELISA method with sheep or goat polyclonal anti-human complement proteins for capture and the same labeled antibody for detection. Results were expressed as the percentage of values obtained in the same experiment with a reference plasma pool prepared from 100 healthy blood donors (normal ranges were 100% ± 30%, as calculated by the results from 50 individual healthy donors).

|  | **Antibodies** | **Company** | | **Reference** | |
| --- | --- | --- | --- | --- | --- |
| AP | Sheep Anti-Human Factor H | The Binding Site |  | PC030 |  |
|  | Sheep Anti-Human Factor I | Abcam |  | ab8843 |  |
|  | Sheep anti-Human Properdin | The Binding Site |  | PC116X |  |
| TP | Sheep Anti-Human C5 | Abcam |  | ab8789 |  |
|  | Goat Anti-Human C6 | ComplementTEch |  | A223 |  |
|  | Goat Anti-Human C7 | ComplementTEch |  | A224 |  |
|  | Goat Anti-Human C8 | Quidel |  | A309 |  |
|  | Goat Anti-Human C9 | Quidel |  | A310 |  |

|  |
| --- |
|  |
|  |
|  |
|  |
|  |

|  |
| --- |

**Supplemental Table 2 : Summary of all genetic changes associated with functional or quantitative deficiency**

| Protein | Gene | Nucleotide change | Variant | Type | GnomAD (allele frequency, %) | Previously reported in patients |
| --- | --- | --- | --- | --- | --- | --- |
| C1QB | *C1QB* | c.268 G>A | p.Gly90Ser | missense | 0.003 | yes |
| C1r | *C1R* | c.1246 C>T | p.Gln416* | Stop gained | not known |  |
| C1r | *C1R* | c.859_878del | p.Phe287Glnfs*38 | small deletion | not known |  |
| C1r | *C1R* | c.367 G>T | p.Glu123* | Stop gained | not known |  |
| C1s | *C1S* | c.632 G>T | p.Gly211Val | missense | not known |  |
| C1s | *C1S* | c.1567 C>T | p.Arg523* | Stop gained | not known | yes |
| C2 | *C2* | c.1482_1483delinsC | p.Ala495Profs*9 | small deletion | not known |  |
| C2 | *C2* | c.841_849+19del; p.(Val281_Arg283del) |  | small deletion | 0.4764 | yes |
| C2 | *C2* | c.538 ins GA | p.Asn180Argfs*75 | Insertion | not known |  |
| C2 | *C2* | c.565 C>T | p.Arg189Trp | missense | 0,00085 |  |
| C2 | *C2* | c.1523 G> T | p.Cys508Phe | missense | 0,00086 |  |
| C2 | *C2* | c.1754 C>T | p.Thr585Met | missense | 0,00086 |  |
| C2 | *C2* | c.2200 C>T | p.Arg734Cys | missense | 0,21 |  |
| C2 | *C2* | c.887 T>A | p.Ile296Asn | missense | not known |  |
| C2 | *C2* | c.1930 C>T | p.Gln644X | Stop gained | not known |  |
| C2 | *C2* | c.2005 C>T | p.Gln669X | Stop gained | not known |  |
| C2 | *C2* | c.616+1 G>A |  | Splice | not known |  |
| C2 | *C2* | c.1903-2 A>G |  | Splice | not known |  |
| C3 | *C3* | c.2002del | p.Arg668Alafs*36 | small deletion | not known |  |
| C3 | *C3* | c.2572_2578dup | p.Leu860Profs*66 | Duplication | not known |  |
| C3 | *C3* | c.2290 C>T | p.Arg764* | Stop gained | 0,00082 |  |
| C3 | *C3* | c.4893 G>A | p.Trp1631 Stop | Stop gained | not known |  |
| C3 | *C3* | c.4121-2 A>G |  | Splice | not known |  |
| C5 | *C5* | c.960_962delCAA | p.Asn320del | small deletion | 0,0029 | yes |
| C5 | *C5* | c.2562+3_2562+10del AAGTAGGT |  | small deletion | not known |  |
| C5 | *C5* | c.713 T>C | p.Ile238Thr | missense | 0,009 |  |
| C5 | *C5* | c.754 G>A | p.Ala252Thr | missense | 0,06 | yes |
| C5 | *C5* | c.3033G>C | Glu1011Asp | missense | 0,11 |  |
| C5 | *C5* | c.393 C>A | p.Asp131Glu | missense | not known |  |
| C5 | *C5* | c.421 G>C | p.Val141Leu | missense | not known |  |
| C5 | *C5* | c.4426 C>T | p.Arg1476* | Stop gained | 0,007 | yes |
| C5 | *C5* | c.55 C>T | p.Gln19* | Stop gained | 0,01 | yes |
| C5 | *C5* | c.1356 C>G | p.Tyr452* | Stop gained | not known |  |
| C5 | *C5* | c.65+1 G>A |  | Splice | 0,0008 |  |
| C5 | *C5* | c.3154+3 A>T |  | Splice | 0,0031 |  |
| C6 | *C6* | c.1138delC | p.Gln380Serfs*7 | small deletion | 0,069 | yes |
| C6 | *C6* | c.1879 delG | p.Asp627Thrfs*4 | small deletion | 0,09 | yes |
| C6 | *C6* | c.143 G>A | p.Arg48Lys | missense | 0,021 |  |
| C6 | *C6* | c.1563 C>A | p.Cys521*Htz | Stop gained | 0,0008 |  |
| C6 | *C6* | c.1333 C>T | p.Arg445* | Stop gained | 0,0033 |  |
| C6 | *C6* | c.1786 C>T | p.Arg596* | Stop gained | 0,012 |  |
| C6 | *C6* | c.821delA | p.Gln274Argfs*46 | Splice | 0,049 | yes |
| C6 | *C6* | c.2381+2T>C (IVS16+2) |  | Splice | 0,22 | yes |
| C6 | *C6* | c.301-1G>A (IVS4-1) |  | Splice | not known |  |
| C7 | *C7* | c.1924_1925delAG | p.His643Profs*10 | small deletion | 0,008 | yes |
| C7 | *C7* | c.405delT | p.Asn136Thrfs*2 | small deletion | 0,02 | yes |
| C7 | *C7* | c.479delG | p.Ser160Ilefs*20 | small deletion | not known |  |
| C7 | *C7* | c.633_643del | p.Ser212Hisfs*4 | small deletion | not known | yes |
| C7 | *C7* | c.1410delG | p.Thr471Profs*22 | small deletion | not known |  |
| C7 | *C7* | c.1743delT | p.Phe581Leufs*15 | small deletion | not known | yes |
| C7 | *C7* | c.189 T>G | p.Cys63Trp | missense | 0,0008 | yes |
| C7 | *C7* | c.1135 G>C | p.Gly379Arg | missense | 0,013 | yes |
| C7 | *C7* | c.1561 C>A | p.Arg521Ser | missense | 0,23 | yes |
| C7 | *C7* | c.928 G>A | p.Gly310Arg | missense | 0.005798 |  |
| C7 | *C7* | c.1117 G>A | p.Gly373Arg | missense | not known |  |
| C7 | *C7* | c.193 G>T | p.Gly65* | Stop gained | not known |  |
| C7 | *C7* | c.2107 C>T | p.Gln703* | Stop gained | not known | yes |
| C7 | *C7* | c.2350+1del G (IVS17+1) |  | Splice | 0,00089 |  |
| C7 | *C7* | c.280+1G>A (IVS4+1) |  | Splice | 0,0024 |  |
| C7 | *C7* | c.281-1G>T (IVS5-1) |  | Splice | 0,01 | yes |
| C7 | *C7* | c.2350+2T>C (IVS17+2) |  | Splice | 0,028 | yes |
| C8β | *C8B* | c.249G>T | p.Arg83Ser | missense | 0,0057 |  |
| C8β | *C8B* | c.850C>T | p.Arg284* | Stop gained | 0,0008 | yes |
| C8β | *C8B* | c.271C>T | p.Gln91* | Stop gained | 0,0065 | yes |
| C8β | *C8B* | c.361C>T | p.Arg121* | Stop gained | 0,01 | yes |
| C8β | *C8B* | c.1282C>T | p.Arg428* | Stop gained | 0,11 | yes |
| C8β | *C8B* | c.1105 +1G>A (IVS7+1) |  | Splice | not known |  |
| C8β | *C8B* | c.138delC | p.Phe47LeufsTer14 | Small deletion | 0.0007962 |  |
| C9 | *C9* | c.721del | p.Leu241* | Stop gained | 0,013 |  |
| C9 | *C9* | c.162 C>A | p.Cys54* | Stop gained | 0,098 | yes |
| C9 | *C9* | c.1240+5G>A (IVS8+5) |  | Splice | 0,0049 |  |
| Factor H | *CFH* | c.3693_3696 delATAG | p.X1232I fsX38 | small deletion | not known | yes |
| Factor H | *CFH* | c.157 C>T | p.Arg53Cys | missense | 0,00165 | yes |
| Factor H | *CFH* | c.1291 T>A | p.C431S | missense | 0.00041 | yes |
| Factor H | *CFH* | c.1789 T>C | p.C597R | missense | not known | yes |
| Factor H | *CFH* | c.2018G>C | p.C673S | missense | not known | yes |
| Factor H | *CFH* | c.2697T>A | p.Y899X | Stop gained | not known | yes |
| Factor H | *CFH* | c.791-2A>C (IVS 7-2) |  | Splice | not known |  |
| Factor I | *CFI* | c.1015 C>T | p.Arg339* | Stop gained | 0,0024 |  |
| Factor I | *CFI* | c.1019 T>C | p.Ile340Thr | missense | 0,007 | yes |
| Factor I | *CFI* | c.1071 T>G | p.Ile357Met | missense | 0.003 |  |
| Factor I | *CFI* | c.1108 A>C | p.Ile370Leu | missense | not known |  |
| Factor I | *CFI* | c.1170 C>A | p.Tyr390* | Stop gained | not known |  |
| Factor I | *CFI* | c.1399 T>C | p.Cys467Arg | missense | not known |  |
| Factor I | *CFI* | c.1420 C>T | p.Arg474* | Stop gained | 0,005 |  |
| Factor I | *CFI* | c.1429+1G>C (IVS11+1) |  | Splice | 0,0033 |  |
| Factor I | *CFI* | c.1484 G>A | p.Cys495Tyr | missense | not known |  |
| Factor I | *CFI* | c.1571 A>T | p.D524Val | missense | not known | yes |
| Factor I | *CFI* | c.1719dup p.Gly574Argfs*14 |  | Duplication | not known |  |
| Factor I | *CFI* | c.208_209insT p.Asn70Ilefs*9 |  | Insertion | not known |  |
| Factor I | *CFI* | c.454 G>A | p.Val152Met | missense | 0,00247 | yes |
| Factor I | *CFI* | c.482+1G>C |  | Splice | not known |  |
| Factor I | *CFI* | c.485 G>A | p.Gly162Asp | missense | 0,0008 | yes |
| Factor I | *CFI* | c.559 C>T | p.Arg187* | Stop gained | 0,0016 | yes |
| Factor I | *CFI* | c.772 G>A | p.Ala258Thr | missense | 0,01 | yes |
| Factor I | *CFI* | c.865 G>C | p.Asp289His | missense | not known | yes |
| Properdin | *CFP* | c.146del p.Gly49Valfs*43 |  | small deletion | 0.0034 | yes |
| Properdin | *CFP* | c.819_825dupTGGCCTG p.Gly276Trpfs*83 |  | Duplication | not known |  |
| Properdin | *CFP* | c.1348dupG p.Glu450Glyfs*16 |  | Duplication | not known |  |
| Properdin | *CFP* | c.304 C>T | p.Arg102Trp | missense | 0.00057 |  |
| Properdin | *CFP* | c.730 G>A | p.Glu244Lys | missense | not known |  |
| Properdin | *CFP* | c.844 C>G | p.Arg282Gly | missense | not known |  |
| Properdin | *CFP* | c.961 T>G | p.Trp321Gly | missense | not known | yes |
| Properdin | *CFP* | c.962 G>T | p.Trp321Leu | missense | not known | yes |
| Properdin | *CFP* | c.235 C>T | p.Arg79* | Stop gained | not known | yes |
| Properdin | *CFP* | c.559 C>T | p.Gln187X | Stop gained | 0.00057 | yes |
| Factor D | *CFD* | c.677-678delinsTTCT |  | Del/Ins |  |  |
| Factor D | *CFD* | c.653T>C | p.Leu218Pro | missense |  |  |

**Supplemental table 3: Bioinformatic prediction of the missense variants**

|  |  |  |  |  |  |
| --- | --- | --- | --- | --- | --- |
| Gene | Mutation designation | SIFT | MUTATION TASTER | POLYPHEN-2 | Previously reported |
| C1QB | c.268G>A p.Gly90Ser | Deleterious (score: 0) | Disease causing (p-value: 1) | Probably damaging | yes(1). |
| C1S | c.632G>T p.Gly211Val | Deleterious (score: 0) | Disease causing (p-value: 1) | Probably damaging |  |
| C2 | c.565C>T p.Arg189Trp | Deleterious (score: 0) | - | Probably damaging |  |
| C2 | c.887T>A p.Ile296Asn | Deleterious (score: 0) | - | Probably damaging |  |
| C2 | c.1523 G> T p.Cys508Phe | Deleterious (score: 0) | - | Probably damaging |  |
| C2 | c.1754 C>T p.Thr585Met | Deleterious (score: 0) | - | Probably damaging |  |
| C2 | c.2200C>T p.Arg734Cys | Deleterious (score: 0) | - | Probably damaging |  |
| C5 | c.393C>A p.Asp131Glu | Deleterious (score: 0) | Disease causing (p-value: 0.998) | Probably damaging |  |
| C5 | c.421G>C p.Val141Leu | Deleterious (score: 0) | Disease causing (p-value: 1) | Probably damaging |  |
| C5 | c.754G>A p.Ala252Thr | Tolerated (score: 0.09) | Disease causing (p-value: 1) | Probably damaging | yes(2). |
| C5 | c.713T>C Ile238Thr | Deleterious (score: 0.01) | Disease causing (p-value: 1 | Probably damaging |  |
| C6 | c.143G>A p.Arg48Lys | Deleterious (score: 0) | Disease causing (p-value: 1) | Probably damaging |  |
| C7 | c.189T>G p.Cys63Trp | Deleterious (score: 0) | Disease causing (p-value: 1) | Probably damaging | yes(3). |
| C7 | c.928G>A p.Gly310Arg Htz | Deleterious (score: 0) | Disease causing (p-value: 1) | Probably damaging |  |
| C7 | c.1117G>A p.Gly373Arg Hmz | Tolerated (score: 0.45) | Disease causing (p-value: 0.932) | Probably damaging |  |
| C7 | c.1135G>C p.Gly379Arg hmz | Deleterious (score: 0.02) | Disease causing (p-value: 1) | Probably damaging | yes(4). |
| C7 | c.1561C>A p.Arg521Ser ( C7SD) | Deleterious (score: 0) | Disease causing (p-value: 0.013) | Probably damaging | yes(4). |
| C8b | c.249G>T p.Arg83Ser | Deleterious (score: 0) | Disease causing (p-value: 1) | Probably damaging |  |
| Factor H | c.1291T>A p.Cys431Ser | Deleterious (score: 0) | Disease causing (p-value: 0.985) | Probably damaging | yes(5). |
| Factor H | c.157C>T p.Arg53Cys | Deleterious (score: 0.01) | Disease causing (p-value: 0.956) | Probably damaging | yes(6). |
| Factor H | c.1789T>C p.Cys597Arg | Deleterious (score: 0) | Disease causing (p-value: 1) | Probably damaging | yes(7). (8). |
| Factor H | c.2018G>C p.Cys673Ser | Deleterious (score: 0) | Disease causing (p-value: 0.995) | Probably damaging | yes(7). (8). |
| Factor I | c.454G>A p.Val152Met | Deleterious (score: 0.01) | - | Probably damaging | yes(9). |
| Factor I | c.485G>A p.Gly162Asp | Tolerated (score: 0.49) | - | Probably damaging | yes(9). |
| Factor I | c.772G>A p.Ala258Thr | Tolerated (score: 0.57) | - | Probably damaging | yes(9). |
| Factor I | c.865 p.Asp289His | Deleterious (score: 0) | - | Probably damaging | yes(10). |
| Factor I | c.1019T>C p.Ile340Thr | Deleterious (score: 0.04) | - | Probably damaging | yes(11). |
| Factor I | c.1071 p.Ile357Met | Deleterious (score: 0.02) | - | Probably damaging |  |
| Factor I | c.1108A>C p.Ile370Leu | Tolerated (score: 0.23) | - | Probably damaging |  |
| Factor I | c.1399T>C p.Cys467Arg | Deleterious (score: 0) | - | Probably damaging |  |
| Factor I | c.1484G>A p.Cys495Tyr | Deleterious (score: 0) | - | Probably damaging |  |
| Factor I | c.1571 A>T p.D524Val | Tolerated (score: 0.34) | - | Probably damaging | yes(12). |
| Properdin | c.304C>T p.Arg102Trp | Deleterious (score: 0) | Disease causing (p-value: 1) | Probably damaging |  |
| Properdin | c.730G>A p.Glu244Lys | Deleterious (score: 0) | Polymorphism (p-value: 0.977) | Probably damaging | yes (13). |
| Properdin | c.844C>G p.Arg282Gly | Deleterious (score: 0) | Disease causing (p-value: 0.998) | Probably damaging |  |
| Properdin | c.961T>G p.Trp321Gly | Deleterious (score: 0) | Disease causing (p-value: 1) | Probably damaging | yes(14). |
| Properdin | c.962G>T p.Trp321Leu | Deleterious (score: 0) | Disease causing (p-value: 1) | Probably damaging | yes(15). |

References

1. Roumenina LT, Sene D, Radanova M, Blouin J, Halbwachs-Mecarelli L, Dragon-Durey MA, et al. Functional complement C1q abnormality leads to impaired immune complexes and apoptotic cell clearance. *J Immunol*. (2011) 187:4369-4373.
2. Owen EP, Wurzner R, Leisegang F, Rizkallah P, Whitelaw A, Simpson J, et al. A complement C5 gene mutation, c.754G>A:p.A252T, is common in the Western Cape, South Africa and found to be homozygous in seven percent of Black African meningococcal disease cases. *Mol Immunol*. (2015) 64:170-176.
3. Rameix-Welti MA, Regnier CH, Bienaime F, Blouin J, Schifferli J, Fridman WH, et al. Hereditary complement C7 deficiency in nine families: subtotal C7 deficiency revisited. *Eur J Immunol*. (2007) 37:1377-1385.
4. Fernie BA, Wurzner R, Orren A, Morgan BP, Potter PC, Platonov AE, et al. Molecular bases of combined subtotal deficiencies of C6 and C7: their effects in combination with other C6 and C7 deficiencies. *J Immunol*. (1996) 157:3648-3657.
5. Dragon-Durey MA, Fremeaux-Bacchi V, Loirat C, Blouin J, Niaudet P, Deschenes G, et al. Heterozygous and homozygous factor h deficiencies associated with hemolytic uremic syndrome or membranoproliferative glomerulonephritis: report and genetic analysis of 16 cases. *J Am Soc Nephrol*. (2004) 15:787-795.
6. Merinero HM, Garcia SP, Garcia-Fernandez J, Arjona E, Tortajada A, Rodriguez de Cordoba S. Complete functional characterization of disease-associated genetic variants in the complement factor H gene. *Kidney Int*. (2018) 93:470-481.
7. Ault BH, Schmidt BZ, Fowler NL, Kashtan CE, Ahmed AE, Vogt BA, et al. Human factor H deficiency. Mutations in framework cysteine residues and block in H protein secretion and intracellular catabolism. *J Biol Chem*. (1997) 272:25168-25175.
8. Servais A, Noel LH, Dragon-Durey MA, Gubler MC, Remy P, Buob D, et al. Heterogeneous pattern of renal disease associated with homozygous Factor H deficiency. *Hum Pathol*. (2011).
9. Kavanagh D, Yu Y, Schramm EC, Triebwasser M, Wagner EK, Raychaudhuri S, et al. Rare genetic variants in the CFI gene are associated with advanced age-related macular degeneration and commonly result in reduced serum factor I levels. *Hum Mol Genet*. (2015) 24:3861-3870.
10. Nita IM, Genel F, Nilsson SC, Smart J, Truedsson L, Choo S, et al. Molecular characterization of two novel cases of complete complement inhibitor Factor I deficiency. *Mol Immunol*. (2011) 48:1068-1072.
11. Kavanagh D, Richards A, Noris M, Hauhart R, Liszewski MK, Karpman D, et al. Characterization of mutations in complement factor I (CFI) associated with hemolytic uremic syndrome. *Mol Immunol*. (2008) 45:95-105.
12. Fremeaux-Bacchi V, Dragon-Durey MA, Blouin J, Vigneau C, Kuypers D, Boudailliez B, et al. Complement factor I: a susceptibility gene for atypical haemolytic uraemic syndrome. *J Med Genet*. (2004) 41:e84.
13. Pedersen DV, Roumenina L, Jensen RK, Gadeberg TA, Marinozzi C, Picard C, et al. Functional and structural insight into properdin control of complement alternative pathway amplification. *Embo J*. (2017).
14. Truedsson L, Westberg J, Fredrikson GN, Sjoholm AG, Kuijper EJ, Fijen CA, et al. Human properdin deficiency has a heterogeneous genetic background. *Immunopharmacology*. (1997) 38:203-206.
15. van den Bogaard R, Fijen CA, Schipper MG, de Galan L, Kuijper EJ, Mannens MM. Molecular characterisation of 10 Dutch properdin type I deficient families: mutation analysis and X-inactivation studies. *Eur J Hum Genet*. (2000) 8:513-518.
