# Supplementary material for: Clinical and Genetic Spectrum of a Large Cohort With Total and Sub-total Complement Deficiencies
Source: Front Immunol. 2019 Aug 8;10:1936. doi: 10.3389/fimmu.2019.01936 (PMC6694794; doi:10.3389/fimmu.2019.01936)
Supplement: Supplementary file 2 [file Presentation_1.PPTX]

## Slide 1
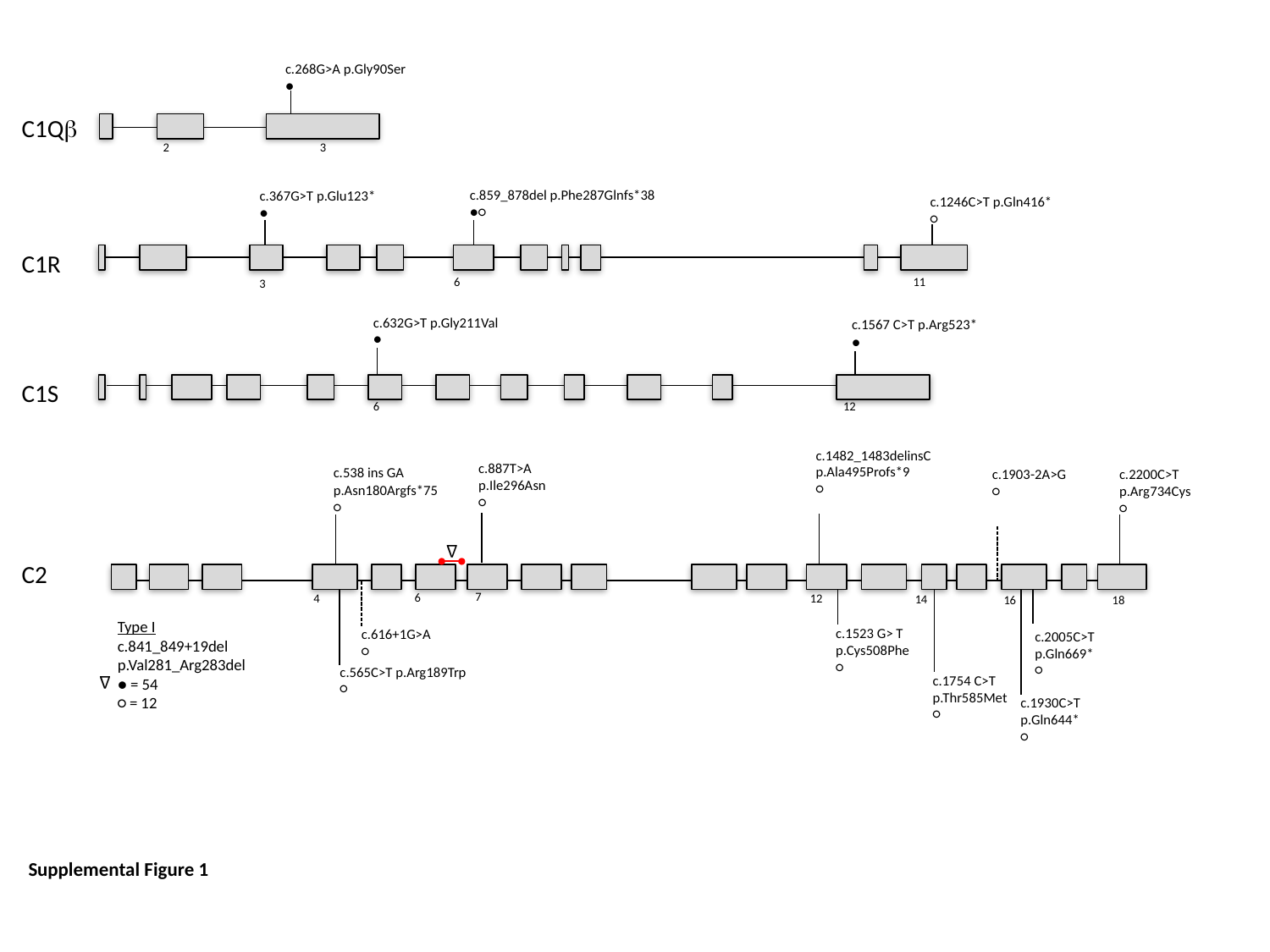

c.268G>A p.Gly90Ser
●
2
3
C1Qb
c.859_878del p.Phe287Glnfs*38
●○
c.367G>T p.Glu123*
●
c.1246C>T p.Gln416*
○
11
6
3
C1R
c.632G>T p.Gly211Val
●
c.1567 C>T p.Arg523*
●
6
12
C1S
c.1482_1483delinsC p.Ala495Profs*9
○
c.887T>A p.Ile296Asn
○
c.538 ins GA p.Asn180Argfs*75
○
c.2200C>T p.Arg734Cys
○
c.1903-2A>G
○
Δ
7
6
12
4
14
16
18
Type I
c.841_849+19del p.Val281_Arg283del
● = 54
○ = 12
c.1523 G> T p.Cys508Phe
○
c.616+1G>A
○
c.2005C>T p.Gln669*
○
c.565C>T p.Arg189Trp
○
c.1754 C>T p.Thr585Met
○
Δ
c.1930C>T p.Gln644*
○
C2
Supplemental Figure 1

## Slide 2
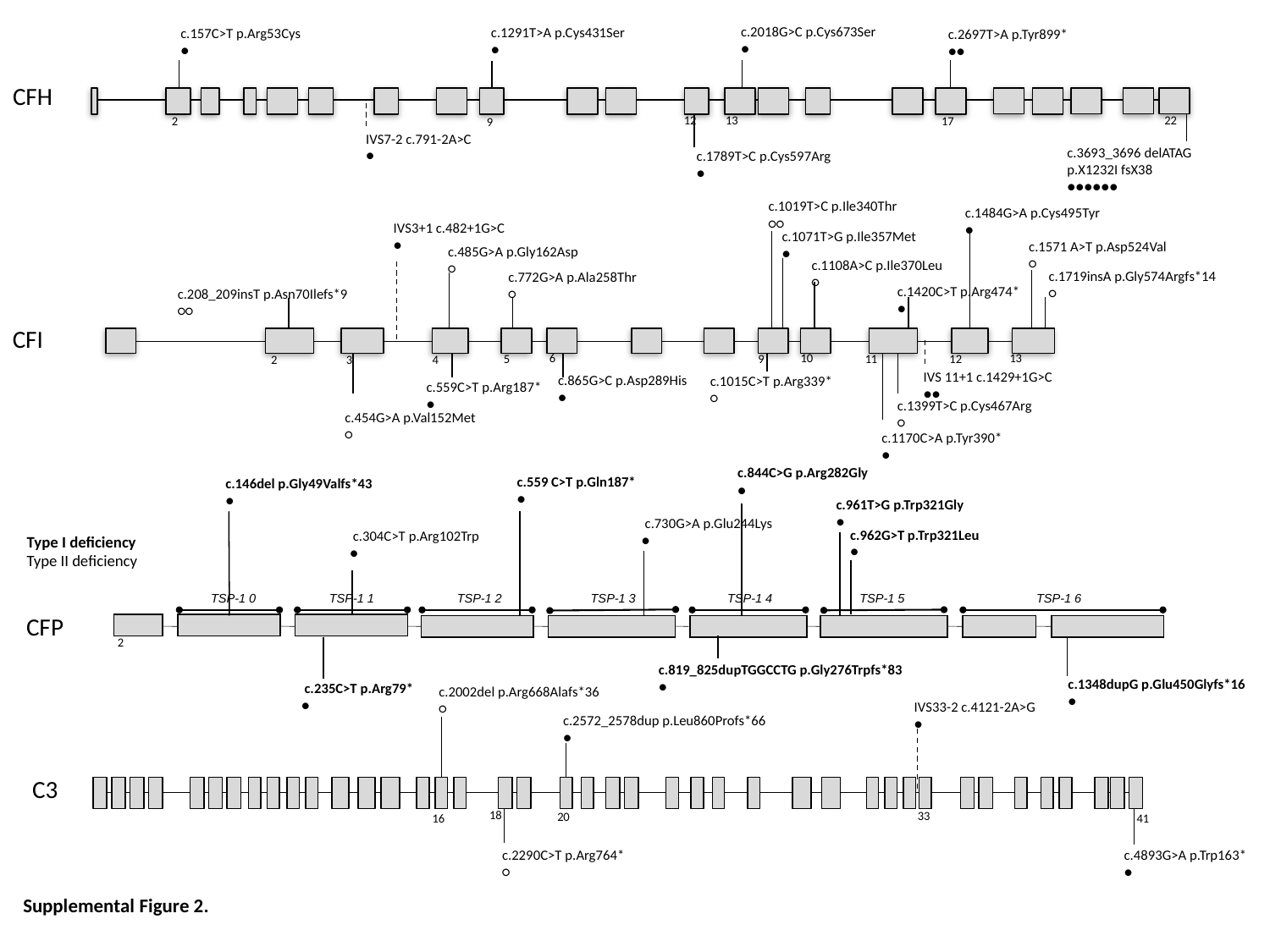

c.2018G>C p.Cys673Ser
●
c.1291T>A p.Cys431Ser
●
c.157C>T p.Arg53Cys
●
c.2697T>A p.Tyr899*
●●
13
12
17
2
9
c.1789T>C p.Cys597Arg
●
c.3693_3696 delATAG p.X1232I fsX38
●●●●●●
22
CFH
IVS7-2 c.791-2A>C
●
c.1019T>C p.Ile340Thr
○○
c.1484G>A p.Cys495Tyr
●
IVS3+1 c.482+1G>C
●
c.1071T>G p.Ile357Met
●
c.1571 A>T p.Asp524Val
○
c.485G>A p.Gly162Asp
○
c.1108A>C p.Ile370Leu
○
c.1719insA p.Gly574Argfs*14
○
c.772G>A p.Ala258Thr
○
c.1420C>T p.Arg474*
●
c.208_209insT p.Asn70Ilefs*9
○○
13
6
10
9
5
12
11
3
4
2
IVS 11+1 c.1429+1G>C
●●
c.865G>C p.Asp289His
●
c.1015C>T p.Arg339*
○
c.559C>T p.Arg187*
●
c.1399T>C p.Cys467Arg
○
c.454G>A p.Val152Met
○
c.1170C>A p.Tyr390*
●
CFI
c.844C>G p.Arg282Gly
●
c.559 C>T p.Gln187*
●
c.146del p.Gly49Valfs*43
●
c.961T>G p.Trp321Gly
●
 c.730G>A p.Glu244Lys
●
c.962G>T p.Trp321Leu
●
 c.304C>T p.Arg102Trp
●
TSP-1 0
TSP-1 1
TSP-1 2
TSP-1 3
TSP-1 4
TSP-1 5
TSP-1 6
Type I deficiency
Type II deficiency
CFP
2
c.819_825dupTGGCCTG p.Gly276Trpfs*83
●
c.1348dupG p.Glu450Glyfs*16
●
 c.235C>T p.Arg79*
●
c.2002del p.Arg668Alafs*36
○
IVS33-2 c.4121-2A>G
●
c.2572_2578dup p.Leu860Profs*66
●
C3
18
33
20
41
16
c.2290C>T p.Arg764*
○
c.4893G>A p.Trp163*
●
Supplemental Figure 2.

## Slide 3
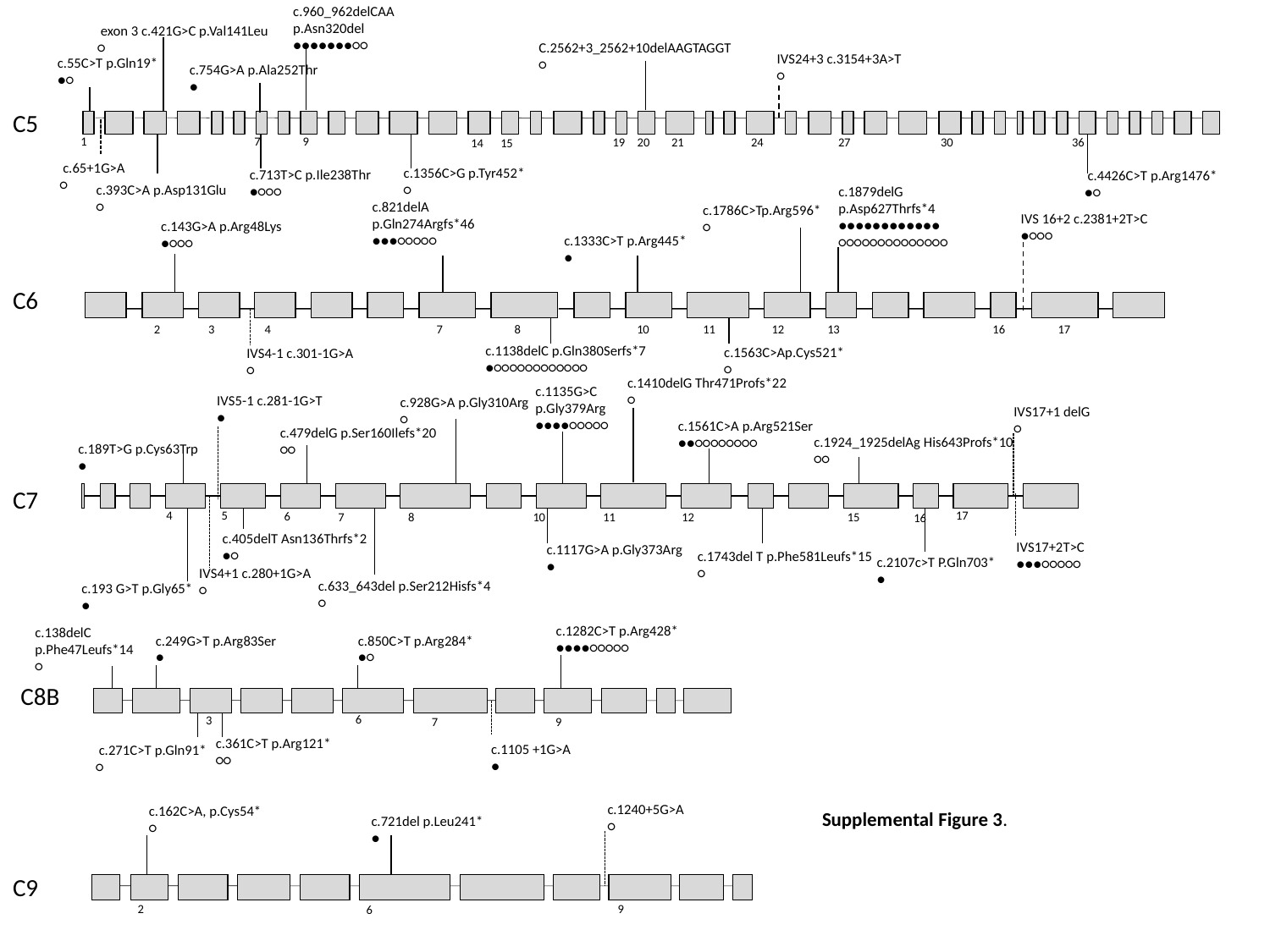

c.960_962delCAA p.Asn320del
●●●●●●●○○
 exon 3 c.421G>C p.Val141Leu
○
c.55C>T p.Gln19*
●○
c.754G>A p.Ala252Thr
●
9
7
1
19
20
21
24
27
30
36
14
15
 c.65+1G>A
○
c.713T>C p.Ile238Thr
●○○○
 c.4426C>T p.Arg1476*
●○
c.393C>A p.Asp131Glu
○
C.2562+3_2562+10delAAGTAGGT
○
IVS24+3 c.3154+3A>T
○
C5
c.1356C>G p.Tyr452*
○
c.1879delG
p.Asp627Thrfs*4
●●●●●●●●●●●●
○○○○○○○○○○○○○○
c.821delA
p.Gln274Argfs*46
●●●○○○○○
c.1786C>Tp.Arg596*
○
IVS 16+2 c.2381+2T>C
●○○○
c.143G>A p.Arg48Lys
●○○○
c.1333C>T p.Arg445*
●
2
3
4
7
8
10
11
12
13
16
17
c.1138delC p.Gln380Serfs*7
●○○○○○○○○○○○○
c.1563C>Ap.Cys521*
○
C6
IVS4-1 c.301-1G>A
○
c.1410delG Thr471Profs*22
○
c.1135G>C p.Gly379Arg
●●●●○○○○○
IVS5-1 c.281-1G>T
●
c.928G>A p.Gly310Arg
○
IVS17+1 delG
○
c.1561C>A p.Arg521Ser
●●○○○○○○○○
c.479delG p.Ser160Ilefs*20
○○
c.1924_1925delAg His643Profs*10
○○
c.189T>G p.Cys63Trp
●
4
5
17
6
7
11
15
10
8
12
16
c.405delT Asn136Thrfs*2
●○
IVS17+2T>C
●●●○○○○○
c.1117G>A p.Gly373Arg
●
c.2107c>T P.Gln703*
●
c.633_643del p.Ser212Hisfs*4
○
c.193 G>T p.Gly65*
●
C7
c.1743del T p.Phe581Leufs*15
○
IVS4+1 c.280+1G>A
○
c.1282C>T p.Arg428*
●●●●○○○○○
c.249G>T p.Arg83Ser
●
c.850C>T p.Arg284*
●○
6
3
9
7
c.361C>T p.Arg121*
○○
c.1105 +1G>A
●
 c.271C>T p.Gln91*
○
c.138delC p.Phe47Leufs*14
○
C8B
c.1240+5G>A
○
c.162C>A, p.Cys54*
○
c.721del p.Leu241*
●
2
9
6
Supplemental Figure 3.
C9

## Slide 4
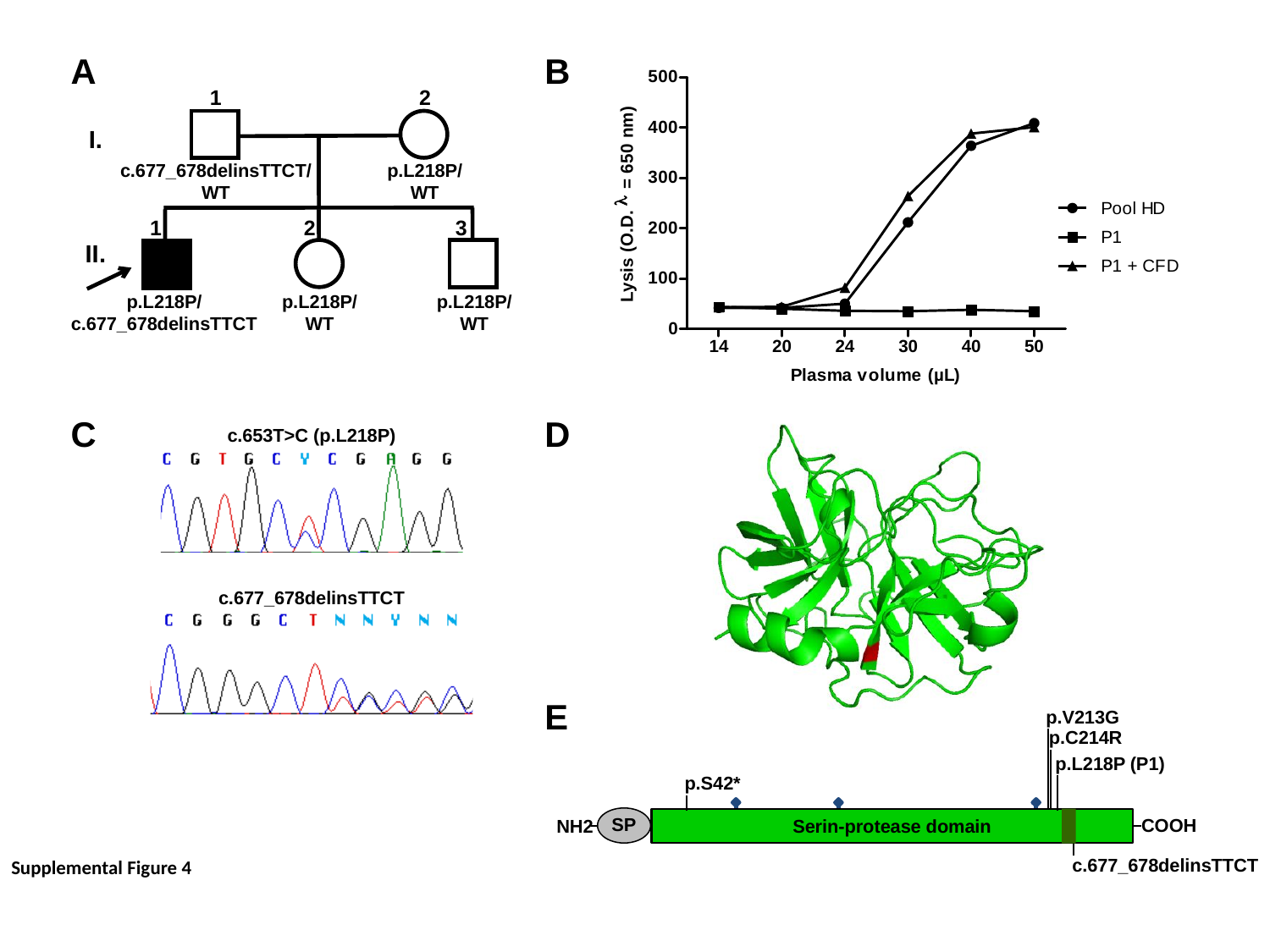

A
B
1
2
I.
c.677_678delinsTTCT/
WT
p.L218P/
WT
1
2
3
II.
p.L218P/
c.677_678delinsTTCT
p.L218P/
WT
p.L218P/
WT
C
D
c.653T>C (p.L218P)
c.677_678delinsTTCT
E
p.V213G
p.C214R
p.L218P (P1)
p.S42*
SP
COOH
NH2
Serin-protease domain
c.677_678delinsTTCT
Supplemental Figure 4
